# Optimising decentralisation for the health sector by exploring the synergy of decision space, capacity and accountability: insights from the Philippines

**DOI:** 10.1186/s12961-018-0402-1

**Published:** 2019-01-10

**Authors:** Harvy Joy Liwanag, Kaspar Wyss

**Affiliations:** 1 Swiss Tropical and Public Health Institute, Basel, Switzerland; 2 University of Basel, Basel, Switzerland; 3 Ateneo de Manila University School of Medicine and Public Health, Metro Manila, Philippines

**Keywords:** Decentralisation, Delivery of healthcare, Health policy, Philippines, Public health administration, Qualitative research

## Abstract

**Background:**

Several studies on decentralisation have used the ‘decision space’ approach to assess the breadth of space made available to decision-makers at lower levels of the health system. However, in order to better understand how decentralisation becomes effective for the health sector, analysis should go beyond assessing decision space and include the dimensions of capacity and accountability. Building on Bossert’s earlier work on the synergy of these dimensions, we analysed decision-making in the Philippines where governmental health services have been devolved to local governments since 1992.

**Methods:**

Using a qualitative research design, we interviewed 27 key decision-makers at different levels of the Philippine health system and representing various local settings. We explored their perspectives on decision space, capacities and accountability in the health sector functions of planning, financing and budget allocation, programme implementation and service delivery, management of facilities, equipment and supplies, health workforce management, and data monitoring and utilisation. Analysis followed the Framework Method.

**Results:**

Across all functions, decision space for local decision-makers was assessed to be moderate or narrow despite 25 years of devolution. To improve decision-making in these functions, adjustments in local capacities should include, at the individual level, skills for strategic planning, management, priority-setting, evidence-informed policy-making and innovation in service delivery. At institutional levels, these desired capacities should include having a multi-stakeholder approach, generating revenues from local sources, partnering with the private sector and facilitating cooperation between local health facilities. On the other hand, adjustments in accountability should focus on the various mechanisms that can be enforced by the central level, not only to build the desired capacities and augment the inadequacies at local levels, but also to incentivise success and regulate failure by the local governments in performing the functions transferred to them.

**Conclusion:**

To optimise decentralisation for the health sector, widening decision spaces for local decision-makers must be accompanied by the corresponding adjustments in capacities and accountability for promoting good decision-making at lower levels in the decentralised functions. Analysing the health system through the lens of this synergy is useful for exploring concrete policy adjustments in the Philippines as well as in other settings.

**Electronic supplementary material:**

The online version of this article (10.1186/s12961-018-0402-1) contains supplementary material, which is available to authorized users.

## Background

Decentralisation could be defined as the transfer of decision-making authority from higher to lower levels of administration [1, 2]. In the Philippines, one major motivation for pursuing decentralisation, not only of local health services but also of other services (e.g. agriculture, infrastructure, social welfare, tourism, etc.), was to empower communities to address their own needs by bringing decision-making closer to them [3]. The Philippines decentralised government health services in 1992 through devolution with the implementation of the Local Government Code [4]. At that time, the Philippine Department of Health (DOH), the ministry primarily responsible for the government health sector nationwide, split the administration of health services into the autonomous local governments across the archipelago, currently numbering 81 provinces, with 1490 municipalities and 145 cities within these provinces. Under devolution, the national government, through the DOH, continues to set the national objectives and policies for the health system while implementation and delivery of health services at local levels is the responsibility of the local governments. More specifically, the provinces assumed responsibility for provincial and district hospitals that provide secondary and tertiary care, while municipalities within these provinces became responsible for the Rural Health Units (RHUs) that deliver primary care services. Cities, on the other hand, may own both hospitals and RHUs and provide both levels of care. After 25 years of devolution, opinions about its impact to local health services continue to vary [3, 5–8], and there have been attempts by various political groups to amend the law in order to, on the one hand, reverse devolution and re-centralise health services once more [9–11] or, on the other hand, expand decentralisation even further by changing the current structure of government from a unitary republic into a federal form [12].

This ambivalence in the Philippines on whether or not to decentralise is not unique, and even recent systematic reviews that examined decentralisation in several countries have concluded that the evidence for its effectiveness in improving health system performance is mixed [13–17]. The dearth in the evidence is partly due to the difficulty in measuring health sector decentralisation itself, indeed a complex process which presents in various shapes and sizes, such as devolution, deconcentration or delegation [1, 18, 19], the boundaries of which may not always be clear in any given context. It is therefore not easy to compare decentralisation of health services between different countries or settings without first considering what health sector functions exactly are decentralised, who are the decision-makers transferring and assuming such functions, and at which levels of the health system is decision-making made. Nevertheless, we have previously argued that the complexity of decentralisation is not an excuse to leave it unexamined, especially when it continues to be viewed as a strategy for health sector reform [20]. The tool that has emerged as useful for analysis is the ‘decision space’ approach developed by Bossert [21], which examines the breadth of space (i.e. ‘wide’, ‘moderate’ or ‘narrow’) within which decision-makers are able to make decisions for the functions they have taken on because of decentralisation. Decision space has been used to analyse health sector decentralisation in several countries [22], such as in Ghana and Zambia [23], Colombia and Chile [24], Pakistan [25, 26], Fiji [27], India [28], Uganda [23, 29], Kenya [30–33], Tanzania [34], and the Philippines [23]. We have also previously reported on the conditions that make decentralisation effective in improving the health system in the Philippines based on the decision space approach [35].

### Decision space, capacity and accountability

Many studies that draw from the decision space approach mostly focus on assessing the difference between de jure and de facto decision spaces, and often reach the conclusion that de facto decision space at lower levels remained narrow or moderate despite decentralisation in policy (de jure), which should have granted a wider space. Thus, many studies make the recommendation to widen de facto decision space further in order to truly empower decision-makers at lower levels of the system. However, two papers on Pakistan [25, 26] stand out because these went beyond decision space and explored its synergy with the dimensions of capacity and accountability. Bossert et al. [25] have proposed visualisation of the synergy of these dimensions as three corners of a triangular model whose complementary interactions lead to improved service delivery, and have provided a statistical justification for this synergy by assessing improvements in outcomes of the maternal and child health programme after building the capacities of local decision-makers in several districts in Pakistan [26].

Indeed, capacity-building is a favourite catchphrase in health systems strengthening. Previous studies have already shown a positive link between health system performance and individual [36] and institutional capacities [37]. Capacity-building can also be an overused term that means no more than mere training, unless it is accompanied by a serious attempt to map the capacity components to assess any impact to capacity enhancement [38]. It may also be viewed to include not only the capacitation of individuals and organisations but also the enabling environment that nurtures it [39]. Thus, beyond individual and organisational capacities, the concept of systemic capacity-building has been promoted to put in place structures and processes that support optimal decision-making through time despite changes in personnel or external interference [40].

Like capacity-building, accountability is also a buzzword in health systems, albeit even less tangible a concept than the former. It may be understood to have two general elements; first, providing an account (i.e. information about the situation) and, secondly, holding into account (i.e. a system of rewards and sanctions for performance) [41]. However, the more important questions are ‘who is accountable to who?’ and ‘how is this accountability enforced?’ Understanding accountability therefore necessitates identifying the linkages between system actors and organisations where, on the one hand, too few connections between decision-makers suggest less control that can enable problems like corruption. On the other hand, too many connections may suggest confusion on who should be held responsible [42]. A recent study has also proposed that interpersonal positive interactions are a key to strengthening accountability in health systems when they complement bureaucratic or audit-style accountability mechanisms [43]. Accountability may also be viewed in (although not limited to) the broad categories of financial accountability, which tracks budget allocation and its correct utilisation; performance accountability, which monitors successful attainment of targets that were previously agreed upon; or political accountability, which compels elected governments to fulfil electoral promises, or those appointed to leadership positions to exert a serious effort to address the needs of the people they serve [42].

**Fig. 1:**
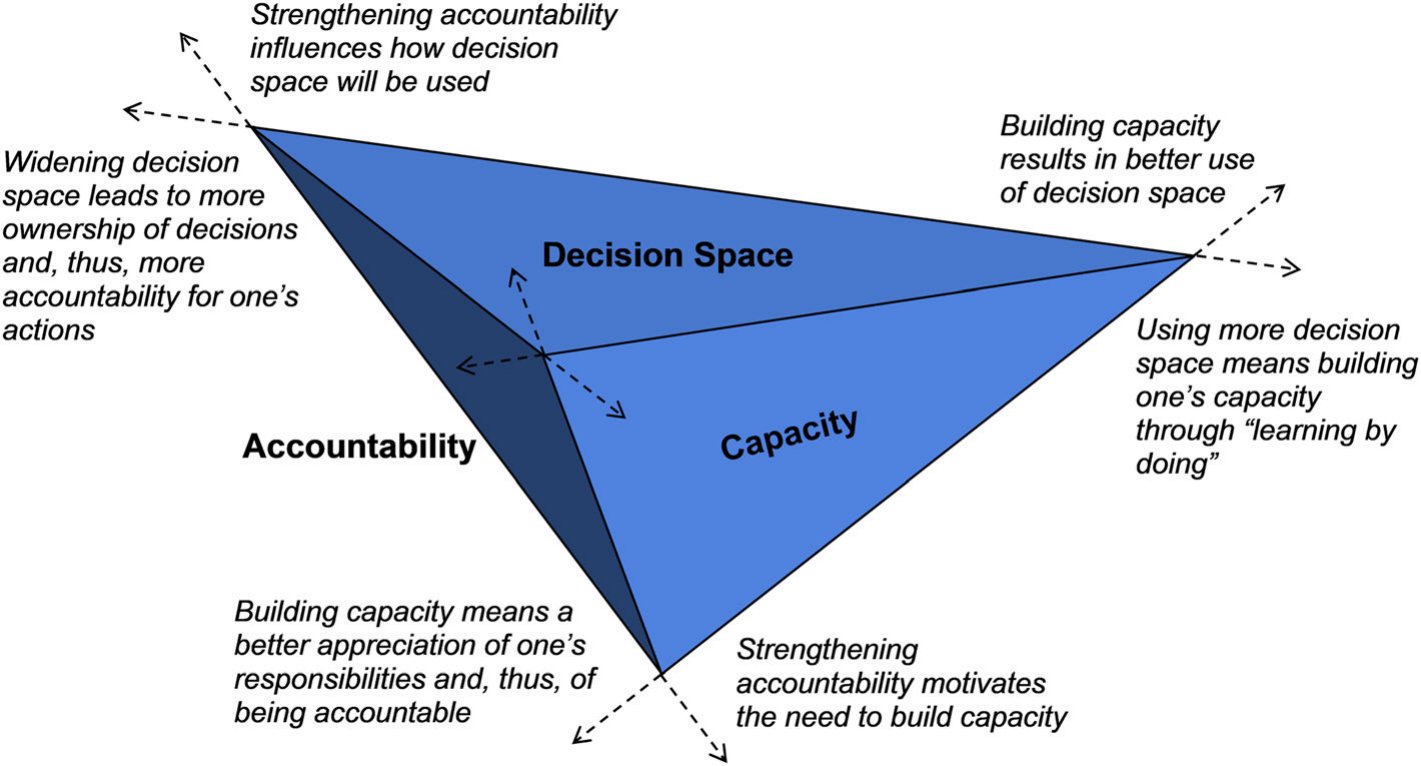
The modified three-dimensional pyramid model for visualising the synergy of decision space, capacity and accountability in the context of health sector decentralisation

Taking into consideration that a synergy means that the interactions of components produce an effect greater than the sum of individual components, we modified Bossert’s earlier model for the synergy into a three-dimensional figure of a pyramid that visualises the three dimensions as a more dynamic and integrated whole (Fig. 1). This modified pyramid model enables better appreciation of the mutually reinforcing interactions between the dimensions, such that the expansion of one contributes to the enhancement of the others. For example, we postulate that, as decision space is widened at local levels through decentralisation, the capacities of local decision-makers would likewise need to be expanded as they perform their new functions and ‘learn by doing’, which would also give them a sense of ownership for their decisions and, thus, a better recognition of their accountability. Similarly, building the capacity of decision-makers would result in a better use of their decision spaces, as well as a better appreciation of their responsibilities, which hold them accountable for the choices they make. Finally, strengthening accountability mechanisms would influence how decision space is used, and would also motivate the need to build the capacities of decision-makers at lower levels of the system.

### Exploring the synergy in the Philippines

Using this synergy as a lens, we aimed to analyse how to optimise decentralisation for the health sector in the Philippines by describing the functions that have been decentralised, examining the decision space available at lower levels for these functions, identifying the capacities of local decision-makers that have to be expanded to carry out these functions well, and exploring the accountability mechanisms that the central level could enforce to ensure good decision-making in these functions. We then recommend a number of policy adjustments based on this analysis to optimise the performance of functions at local levels in the devolved Philippine health system.

## Methods

Following a qualitative research design, we developed an interview guide for exploring the activities or tasks where decision-making for the health sector is performed at local levels in the Philippines. Using the first author’s (HJL) personal and professional connections in the Philippines, we then purposively selected and contacted decision-makers who serve (or previously served) in the government health sector and who represent a wide range of local settings. HJL is also a citizen of the Philippines who has been familiar with the country’s health system through his work as an academic researcher. Between January and April 2017, 27 decision-makers were interviewed, up until the point when we judged that saturation had been achieved [44]. The interviews lasted 1 hour and 4 minutes, on average, and were all performed face-to-face. Interview participants were 17 (63%) males and 10 (37%) females who worked in the Philippine government sector for an average of 23.6 years. While the methods of qualitative research did not allow us to obtain a statistically representative sample of informants for this study, purposive selection of participants was undertaken with the aim of maximising the variation in their profiles in terms of current roles and organisational affiliations, levels of decision-making, and geographic locations. A summary of the characteristics of these decision-makers is provided in Table 1.

**Table 1:** Summary of characteristics of the decision-makers interviewed for this study. Additional details on their career history and location of work assignments have been published elsewhere (Liwanag and Wyss [35])

No. of interviewees	27
Males	17
Females	10
Highest educational attainment	
MD plus Master’s degree	17
MD	5
Law degree	3
Master’s degree	1
Bachelor’s degree	1
Average duration of service in the public sector (years)	23.6
Average duration of the interviews (min)	64
Category of current roles	
Career health officers (provincial, municipal and city health officers)	10
DOH directors (national and regional directors)	6
Local politicians	6
Executive of PhilHealth	1
“Doctor to the *Barrio*” (i.e. DOH-hired physician deployed to serve under a local government)	1
Medical school administrator	1
Government hospital administrator	1
Head of an NGO	1
Organisational affiliation at the time of interview	
Local governments	15
DOH	6
NGOs	2
PhilHealth	1
Government hospital	1
Philippine Congress	1
Academe	1
Level of decision-making at the time of interview	
National level	7
Regional level	3
Provincial level	4
City level	3
Municipal level	9
Not applicable	1
Geographic focus of role at the time of interview	
Nationwide	6
Luzon	13
Visayas	1
Mindanao	7

*DOH* Department of Health, *MD* Doctor of Medicine, *NGO* non-governmental organisation, *PhilHealth* Philippine Health Insurance Corporation

The proposal for this study was approved in the Philippines by the National Ethics Committee (no. 2016–013) and in Switzerland by the Ethikkommission Nordwest- und Zentralschweiz (no. 2016–00738). This article was written using the guidance provided by the Criteria for Reporting Qualitative Research (COREQ) [45]. All study participants read and signed an informed consent form prior to the interviews, which were audio-recorded and manually transcribed in Microsoft Word 2016. Information on personal identities were replaced with codes in the transcripts, accessible only to the authors to maintain confidentiality. Each transcript was reviewed at least twice while listening to the audio recording to ensure accuracy of transcription and to improve familiarity with the data. Transcripts were loaded into MAXQDA Standard 12 (VERBI GmbH Berlin 2018) for coding and analysis.

Data analysis was based on the Framework Method, as previously described in the literature [46–48]. Building on our previous analysis [35], we combined deductive and inductive approaches in the sense that we began coding using the trio of decision space, capacity and accountability as the initial thematic framework, which was later populated with categories in an iterative fashion as the coding was performed.

### Health sector functions

As gathered from our interviews, we identified six health sector functions, which we defined as broad categories of activities or tasks within which decision-making is performed. The definitions used for these health sector functions were based on definitions used in previous studies on decision space, which we subsequently modified according to the experience in the Philippines. While the boundaries of these functions would overlap in some situations, we nevertheless organised these decentralised functions according to the following:

1. *Planning –* development of plans for local health services in a regular manner, involvement of stakeholders in the planning process, and/or implementation of what has been indicated in these plans;
2. *Financing and Budget Allocation –* allocation of budget (either from national/central or local sources) to support local health services, creation of additional sources of income to finance local health needs, and/or utilisation of the local budget according to what it was intended for;
3. *Programme Implementation and Service Delivery –* implementation of health programmes at local levels following national guidelines, implementation of locally designed services that meet local needs and are suitable to the local context, and/or provision of services that satisfy the standards of quality;
4. *Management of Facilities, Equipment and Supplies –* building the types and quantity of local health facilities in areas where these are needed, maintenance and upgrade of these facilities, and/or providing the equipment and supplies (e.g. medicines) required to make these facilities fully functional;
5. *Health Workforce Management –* hiring (and firing) the cadres and number of health workers required to meet the needs of the local population, providing adequate salaries and benefits for these health workers according to the national standard rates, and/or supporting their training needs and career aspirations;
6. *Data Monitoring and Utilisation –* choosing the indicators for monitoring the performance of local health services, collecting these indicators in an accurate and timely manner, performing data management efficiently, and/or using the collected data to inform decisions at local levels.

**Fig. 2:**
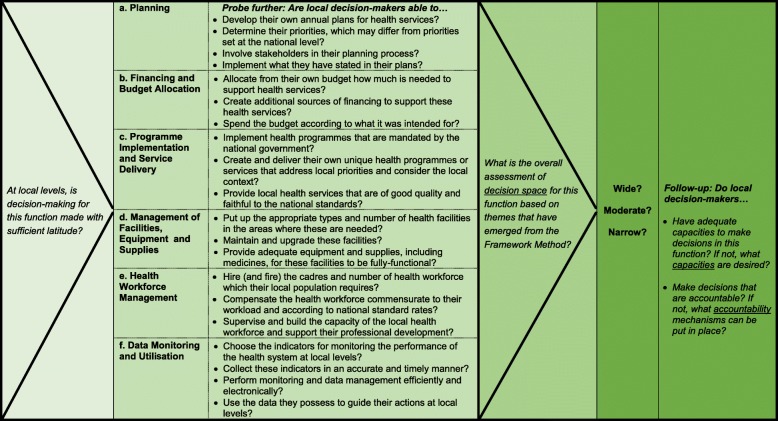
Outline of the guide questions posed during the interviews to explore decision-making in six functions and be able to assess the overall breadth of decision space as wide, moderate or narrow. Follow-up questions related to capacities and accountability are likewise included. An example of the full interview guide from which these questions were taken is available as supporting information in Liwanag and Wyss [35]

Assessment of decision space drew from the information obtained during the in-depth interviews using a list of guide questions that provided flexibility in assessing the decision spaces available to decision-makers in the performance of these functions as wide, moderate or narrow, as well as their desired capacities and accountability mechanisms that influence their decision-making in these functions. An outline of these guide questions is presented in Fig. 2, while an example of the full interview guide is available as a supporting information file in Liwanag and Wyss [35]. However, unlike a quantitative approach that enables assessment of decision space based on scores, our application of a qualitative approach meant that our assessment relied on the various and common themes that emerged as the transcripts were analysed following the Framework Method. To complement Fig. 2 and adapting from Bossert’s earlier criteria for assessing decision space [21], we further provide Table 2 below as a specific criteria for judging the space for each of the health sector functions as wide, moderate or narrow. The Additional file to this article also provides a selection of illustrative quotes for each function together with an explanation on how decision space was finally assessed overall (Additional file 1). Briefly, we assessed decision space for each function as wide if decision-makers at lower levels are able to make decisions with wide latitude, narrow if mostly unable to make decisions, and moderate if somewhere in between. For functions where decision space was assessed as moderate, yet a number of interviews suggested that the space was narrow in certain situations, an assessment of moderate-to-narrow was made. Given the outcome of decision space assessment, we then analysed the desired capacities for local decision-makers, organised into institutional and individual capacities, to be able to perform each function well, and the accountability mechanisms, organised as current and proposed, that can be enforced by the central level to promote good decision-making in each function.

**Table 2:** Criteria used for assessing decision space at local levels for the purpose of qualitative analysis (adapted from Bossert [21])

Health sector function	Indicator	Decision Space		
		Narrow	Moderate	Wide
a. Planning	Local decision-makers prioritise and develop their own health plans	Local planning possible only if with assistance from the central level	Local planning already taking place, but only optimal if accompanied by significant assistance from the central level	Local planning optimal despite minimal involvement of the central level
	Local decision-makers implement the plans that they developed	Implementation possible only with central level support	Implementation being done but only completed if central level support is available	Full implementation possible even without central level support
b. Financing and Budget Allocation	Local decision-makers have their own sources of income to finance health services	Financing mostly dependent on central sources of funds	Mixed financing, such that local sources of financing are augmented with central sources	Financing mostly provided by local sources of funds
	Local decision-makers spend the budget allocated for health services	Spending mostly restricted by guidelines imposed by the central level	Some of the budget controlled by the local level, and some regulated by the central level	Spending mostly follows how local decision-makers wish to use the budget
c. Programme Implementation and Service Delivery	Local decision-makers implement their own health programmes and services	Local programmes and services mostly follow only what is promulgated from the central level	Local programmes and services follow nationally mandated programmes but also include locally initiated and innovative programmes that address local needs	A good number of innovative programmes and services implemented at local levels with little supervision from the central level
	Local decision-makers deliver health services with good quality	Local programmes and services implemented with poor quality	Local programmes and services implemented with good quality when central level provides additional support and training	Local programmes and services implemented with good quality despite minimal central level involvement
d. Management of Facilities, Equipment and Supplies	Local decision-makers put up the number and type of health facilities needed in their areas	Local facilities built and upgraded mostly through central support	Some facilities built and upgraded by the local level but still a large number of constructions or renovations provided by the central level	Local facilities built and upgraded mostly through the local level’s own efforts and resources
	Local decision-makers ensure functionality of these facilities with adequate equipment and supplies	Local facilities mostly rely on central support for equipment and supplies	Mixed, such that equipment and supplies are provided by both the local and central levels	Local facilities adequately equipped and supplied from the local level’s own efforts and resources
e. Health Workforce Management	Local decision-makers hire (and fire) the health workforce needed by the local population	Local levels unable to hire the workforce needed	Local levels able to hire some of the workforce required, but central level augments many vacancies through deployment of its own staff	Local levels able to hire most of the workforce on their own
	Local decision-makers support the career development of the health workforce	Few opportunities at local levels to support the career development of their workforce	While local levels can support the career development of their workforce, a big chunk of training is still provided by the central level	Training and support for the career development of the workforce sufficiently provided by local levels
f. Data Monitoring and Utilisation	Local decision-makers collect the relevant indicators	Data collection delayed and poorly validated, unless the central level requires and enforces it	Local levels collect the data in a timely and accurate manner when assistance is provided by the central level	Timely and accurate data collection despite minimal intervention from the central level
	Local decision-makers use the data to inform actions	Utilisation of the collected data for actions at local levels not practiced	Local levels collect the data, but central level provides guidance on how to use the data	Data clearly used for actions by the local levels themselves

## Results

### Planning

In the Philippines, where local governments (provincial, municipal and city governments) have assumed the ownership of health facilities and management of public health programmes at local levels, decision space in planning for local decision-makers was assessed as moderate. While local governments, which are headed by elected politicians, have long been granted the authority to plan on their own, various experiences in the Philippines suggest that successful planning relies on the local government’s ability to plan well and on the local politician’s (provincial governor or city/municipal mayor) regular convening of the multi-sectoral Local Health Board (LHB), which may or may not meet depending on the politicians' prioritisation of health concerns during their term of office. This is consistent with one previous study concluding that there were more public health initiatives, community consultations and spending for health in local governments with functional LHBs compared to those whose LHBs did not meet regularly [49]. To assist local governments in planning, the DOH, through its regional offices, has also been sponsoring and conducting the annual Investment Planning for Health, a mechanism through which local government health personnel are given technical assistance [50] and trained by the DOH on how to prepare their health plans, identify needs, and request for additional support from the national government for the full implementation of these plans. Given this moderate decision space, decision-making in planning may be enhanced when local decision-makers have adequate capacities for performing strategic planning in a regular and timely manner and for involving multiple stakeholders in the planning process. On the other hand, some of the accountability mechanisms to promote good decision-making in planning include requiring the functionality of the LHB as a condition for local governments to receive additional support from the national/central government, reviewing and approving these plans at central/regional levels to ensure alignment with national objectives, and strict monitoring of the implementation of these plans to ensure satisfactory completion before further support from the national government to these local governments could be provided in the future (Table 3).

**Table 3:** Assessment of decision spaces and the desired adjustments in capacities and accountability mechanisms for the health sector functions of (a) Planning, (b) Financing and Budget Allocation, and (c) Programme Implementation and Service Delivery

Health sector functions, i.e. activities or tasks that involve decision-making	Illustrative quotes^a^	What is the decision space at local levels?	What capacities of local decision-makers are desired?	What accountability mechanisms can be put in place by the national/central level?
Planning	Mayor of a low-income municipality who is also a medical doctor, 26 years in government: “*National government wants LHBs to be functional, but it’s up to us to make it functional. We meet for the municipal health action planning, which flows from the Barangay [village] health action planning. So the municipal plan is a consolidation of the various Barangay plans. The DOH has a representative in the LHB, and that is very good because the mayor does not know everything. It’s a coincidence that the mayor here is a doctor, but how about those areas whose mayor is not a doctor? We need help from the DOH for the technical aspects, for example, in the family planning programme, immunisation,* etc. *We also review our shortcomings. But, you know, it varies from one municipality to another* [laughs]*. That is the disadvantage of devolution, right? The way things are is not uniform and depends on municipal leadership.*”	Moderate	Institutional: • Institutional commitment to perform the planning process regularly • Openness to the participation of multiple stakeholders in the planning process Individual: • Strategic planning skills	Currently in place but may be enhanced: • Technical assistance to local governments for performing planning effectively • Local plans reviewed and approved at central/regional levels to ensure alignment with national objectives • Monitoring by the central/regional levels of local plan implementation Potential policy consideration: • Continuing augmentation for local health services conditional on local government’s regular conduct of planning and satisfactory implementation of previous plans
Financing and Budget Allocation	Provincial Health Officer of a high-income province, 21 years in government: “*About 25–27% of our Internal Revenue Allotment is allocated for our hospitals, and about 5–7% for preventive services. I have an income recovery scheme here. The province provides the budget for maintenance and other operating expenses of hospitals, but I tell the hospitals to recover at least 90% of that and return the funds to the province. The hospitals are able to recover it through their PhilHealth income, and also through income from services not covered by PhilHealth but outpatients pay for, such as ultrasound or CT scan. So majority of our local budget is used for hospital operations, and that’s curative, right? That means we spend so little for preventive services, which should have a bigger investment. This is what I want to ask from DOH, to provide additional funding to enhance our delivery of public health programmes.*”	Moderate-to-narrow	Institutional: • Ability to create alternative income sources (except user fees which may reduce access) that are earmarked for local health services Individual: • Skills for priority-setting, with an emphasis for primary/preventive care • Capacity for evidence-informed, rather than politically motivated, funding decisions	Currently in place but may be enhanced: • Strict implementation of PhilHealth guidelines that limit local governments to use their PhilHealth income exclusively for health-related needs Potential policy consideration: • Accreditation of local health facilities to be eligible for reimbursements from PhilHealth may include a requirement for local governments to provide a minimum allocation (depending on income class) from its own local budget as counterpart to finance local health services
Programme Implementation and Service Delivery	High-level official of the DOH Central Office, 28 years in government: “*As the devolution process evolved, and as local governments become more capable to handle their health services, there were circulars issued by the DOH programmes in the central office to ensure quality, for example, on how to package the tuberculosis control programme for their locality. Some of these guidelines sought to remedy the negative aspects of devolution, and so the concept of interlocal health zones or service delivery networks to group local governments together emerged to encourage different local governments serving the same catchment area to deliver health services in a harmonised manner.*”	Moderate	Institutional: • Willingness to cooperate with neighbouring local governments for a functional service delivery network for sharing of resources and inter-facility patient referrals Individual: • Innovation in the delivery of local health programmes (while maintaining fidelity to national objectives) that address unique health needs and are suitable to the local context	Currently in place but may be enhanced: • Development of technical guidelines that maintain fidelity in the delivery of nationally mandated programmes at local levels • Training of local government staff in implementing these programmes • Strengthening of service delivery networks by strategically grouping local governments together and facilitating their interlinking with one another Potential policy consideration: • Wider recognition and promotion of models of innovative service delivery programmes by local governments

^a^Only a few illustrative quotes could be presented here due to space limitations. Please refer to Additional file 1 for the full list of illustrative quotes, which form the basis of the assessment of decision space and the recommendations for capacity and accountability

*DOH* Department of Health, *LHB* Local Health Board, *PhilHealth* Philippine Health Insurance Corporation

### Financing and budget allocation

Decision space for financing and budget allocation was assessed to be moderate-to-narrow because the flexibility in making local funding decisions in the Philippines largely depends on the income classification of the local government. For instance, local governments in highly urbanised cities get a bigger share (and, thus, more choices for budgeting) of their Internal Revenue Allotment from the national government, which is responsible for pooling tax collection across the country and redistributing the revenue to the various local governments based on a formula that considers local population and land area. Consequently, local governments in smaller provinces or rural municipalities get a smaller share of their Internal Revenue Allotment, which is often insufficient to support labour-intensive health services. As a result, most local governments have relied significantly on payments from the Philippine Health Insurance Corporation (PhilHealth) [51, 52], which administers the national social health insurance programme, to sustain the operations of their health facilities. These observations gathered from the interviews also complement previous studies on the Philippines that noted dampened spending for health by the local governments across the years, especially in provinces [5], as well as the dominance of narrow electoral objectives in influencing financing decisions for health [53]. Decision-making in financing and budget allocation may then be optimised when local decision-makers have adequate capacities for performing priority-setting [54] (including an emphasis for primary/preventive care services) and evidence-informed (rather than politically motivated) funding decisions, as well as for creating alternative sources of income (except user fees that may reduce access) that are earmarked for financing local health services. Accountability mechanisms may include requiring local government-owned health facilities to meet the minimum standards of quality before these are accredited by PhilHealth to become eligible for receiving payments for services provided. PhilHealth accreditation may also include a requirement for the local government to provide a minimum allocation from its own budget as counterpart for financing local health services. Moreover, existing national guidelines on the utilisation of PhilHealth income by local governments should be strictly enforced in order to push local governments to spend the fund exclusively for health-related expenses alone, with future reimbursements from PhilHealth conditional on the local government’s compliance with these guidelines.

### Programme implementation and service delivery

Decision space for programme implementation and service delivery was assessed as moderate considering that local governments in the Philippines are already able to develop and implement their own health programmes, but at the same time mostly relying on the health programmes being promulgated by the DOH from national/regional levels for implementation at local levels. However, the devolved structure of governance may also result in weak implementation of programmes such as what was noted for the malaria control programme in a previous study [55], where ineffective linking between central and local levels resulted in inconsistent implementation by local governments that failed to adhere to the national objectives of the programme. Thus, decision space may be better used when local decision-makers have the capacity for innovation in the delivery of health programmes (while maintaining fidelity to national objectives) that address specific local needs, more appropriate to the culture, and thus more effectively implemented at local levels. Local governments may also be better equipped to perform this function if they have the capacity to cooperate with other neighbouring local governments [6], despite each being distinct political units as a result of devolution, to constitute a functional service delivery network that facilitates coordination of patient referrals, or resource sharing for more efficient delivery of care (e.g. sharing medicines when the health facility of another local government has stockouts, or allowing health professionals to assist temporarily in a neighbouring health facility owned by a different local government that lacks staff). Accountability in this function may be strengthened when national/central decision-makers maintain its responsibility for developing and enforcing the technical guidelines to be complied with by the local governments in the delivery of nationally mandated health programmes (e.g. expanded programme on immunisation, tuberculosis control programme, or non-communicable diseases control programme, etc.), including the training of local government health staff who will carry out these programmes at local levels. The national/central level may also strengthen accountability by using, as an incentive, the recognition and promotion of innovative health programmes developed by some local governments that can be emulated by other local governments, and also by facilitating the grouping of adjacent local governments to constitute functional service delivery networks.

### Management of facilities, equipment and supplies

Decision space for the management of facilities, equipment and supplies was assessed as moderate because local governments already have full management control over health facilities that they own, but nevertheless also continue to depend on continuing assistance from the DOH for the upgrade of their facilities, including the provision of equipment and supplies (e.g. medicines, vaccines, contraceptives, laboratory diagnostic kits, etc.) for use in these facilities. Decision space for this function may be optimised when local decision-makers are equipped with the adequate management skills needed for running health facilities and programmes effectively. Such capacities may in fact already be possessed by the local government health officer, the career health official employed by the local government to manage local health services, but not by the elected governor or mayor who may lack the technical understanding or appreciation of the significance of public health. Capacities for local governments to engage the private sector may also be expanded so that some aspects of service delivery can be made more efficient through public–private partnerships. Examples of such partnerships in selected local governments in the Philippines include outsourcing the provision and maintenance of expensive equipment required by the provincial hospital (e.g. x-ray machine, ultrasound machine, or CT scanner) where the income from the use of the equipment is shared by the local government and the private provider. Another example is the provision of a steady supply of medicines in the local government hospital through a consignment agreement with a private seller, which not only minimises drug stockouts but also enables the local government to pay only for the medicines that are actually used. On the other hand, strengthening accountability in this function may be achieved when central/regional decision-makers strictly enforce licensing of local government health facilities to maintain quality, but at the same time provide technical assistance to those local governments that are struggling to achieve accreditation for their health facilities. Another mechanism is for the central/regional level (i.e. DOH) to perform pooled or central procurement of selected supplies (e.g. medicines and vaccines) on behalf of local governments nationwide for the purpose of maintaining leverage in price negotiation, rather than let each individual local government negotiate on its own. These supplies are then provided as augmentation for local health facilities subject to the local government’s satisfactory utilisation of previous augmentations. In the Philippines, the DOH has also been running a Health Facility Enhancement Programme that allows the use of national/central funds for the construction of new (or upgrade of existing) local government health facilities. However, such central support through the Health Facility Enhancement Programme must require the provision of counterparts from the local government. For example, the DOH may spend for the expansion of a provincial hospital or the renovation of a city or municipal RHU, but the local government that owns it will be required to hire the additional number of health workers needed to fully operate the upgraded facility. Moreover, strengthening current mechanisms for licensing and accreditation of local government health facilities by the DOH and PhilHealth, respectively, may be one way to enhance quality, as a previous attempt that relied on certification alone failed to improve the quality of services in these facilities [56] (Table 4).

**Table 4:** Assessment of decision spaces and the desired adjustments in capacities and accountability mechanisms for the health sector functions of (a) Management of Facilities, Equipment and Supplies, (b) Health Workforce Management, and (c) Data Monitoring and Utilisation

Health sector functions, i.e. activities or tasks that involve decision-making	Illustrative quotes^a^	What is the decision space at local levels?	What capacities of local decision-makers are desired?	What accountability mechanisms can be put in place by the national/central level?
Management of Facilities, Equipment and Supplies	Director in the DOH Central Office, 28 years in government: “*If you would look at how the DOH works with local governments now, it seems that a bulk of our budget actually goes to them. It’s as if it is not devolved. During the last years, DOH upgraded their facilities. DOH is also providing the commodities for the programmes. DOH is giving them the drugs, TB drugs, and now even hypertensive drugs, diabetic drugs. So there is always that question, are we really in a devolved set-up? It has been observed that the local governments really do not have the capacity for health services. I am not saying that this is happening across the country, but in most municipalities and provinces, most especially in the low-income ones, well, even in some first-class provinces. Why? Because the population has increased but there was no increase in the infrastructure and the personnel. That’s why the DOH augments the local governments.*”	Moderate	Institutional: • Creativity in partnering with the private sector to enhance the delivery of care in local health facilities Individual: • Management skills for running health facilities and public health programmes effectively	Currently in place but may be enhanced: • Licensing and accreditation of local health facilities that meet the standards of quality, while supporting those facilities that do not qualify to eventually meet the standards • National/central support for upgrading local health facilities (especially in resource-poor settings to achieve equity) conditional on the local government’s provision of counterpart Potential policy consideration: • Central-procurement of selected equipment and supplies on behalf of local governments to gain leverage in negotiating prices; provision of these resources as augmentation to local governments but conditional on satisfactory utilisation of past augmentations
Health Workforce Management	Provincial Health Officer of a low-income province, 29 years in government: “*A poor province could only afford this much, and cannot provide salaries like a wealthy province could. In my case, my salary is only for a second-class province, because my province has the capacity of only a second-class local government. If the province becomes first-class, then the salaries will go up too. That is why when you compare the salaries in different classes of provinces or municipalities across the country, the rates would be different. I think salaries should be standardised across the country, regardless of where one is serving, because we are all doctors anyway, same with nurses and midwives. We are all health professionals, right?*”	Moderate-to-narrow	Institutional: • Sufficient financial capacity and regulatory authorisation to: ◦ Hire (and fire) the cadres and number of health workers needed to serve the local population ◦ Provide health workers’ full range of salaries and benefits, including security of tenure Individual: • Deeper appreciation at various levels of governance on the important role played by the health workforce at local levels	Currently in place but may be enhanced: • Deployment of centrally hired health workers to local health facilities that lack them, but conditional on the local government’s: ◦ Provision of counterpart support for the deployed health workers ◦ Commitment to eventually allocate the budget required to hire health workers on their own Potential policy considerations: • Central/regional levels to be officially responsible for providing capacity-building of local government health workers across the country • Implementation of a national policy that discourages local health workers form being partisan during local elections
Data Monitoring and Utilisation	Assistant City Health Officer of a highly urbanised city, 22 years in government: “*Perhaps if you ask the DOH, they would tell you that they are having a hard time with the data because of devolution. It takes a long time for us to submit reports to them. Why am I taking a long time? For my part, I am consolidating all of the reports, including those from our hospitals. So, it is difficult, right? Oh, we do our own surveillance and DOH also does its surveillance, that’s why it is difficult. Actually, there are instances when DOH detects cases first before we do. And there was a time also when we detected it first before they did. So before the DOH even learns about it, we already have a report. That is why maybe for them the DOH is saying that it’s more difficult. Because they feel there is an extra step before the data gets to them, and the city still needs to gather the data from all our health centres.*”	Moderate	Institutional: • Systemic capacity for an integrated and harmonised manner of data monitoring and utilisation in spite of decentralisation and the use of interoperable electronic medical records Individual: • Basic knowledge of epidemiology to understand the relevance of the indicators collected • Skills for evidence-informed public health, or for translating data into action at local levels	Currently in place but may be enhanced: • Deployment of centrally hired data collectors to local governments to validate the local data collected and accelerate data transmission to the central level • Maintenance of a central electronic database, into which local governments will be required to transmit data in a timely manner Potential policy consideration: • Publication of rankings of local governments in achieving selected target outcomes

^a^Please refer to Additional file 1 for the full list of illustrative quotes

*DOH* Department of Health

### Health workforce management

Decision space for health workforce management was assessed to be moderate-to-narrow. While local governments already have full control over the management of its health workers, in many rural areas, the local governments are unable to hire the minimum number of health workers they need due to their lack of resources to pay for their salaries or the absence of an incentive for health workers to serve in these far-flung areas [3]. In local governments that are able to hire, the salaries and benefits vary depending on the financial capacity of the local governments, despite an existing law that provides for standard salary rates for health professionals. Many local governments have thus relied on the DOH’s long-established deployment programme of health workers, such as the Doctors to the *Barrios* programme, to augment their health workforce needs. This deployment programme has enabled the DOH to hire physicians, nurses, midwives, dentists and medical technologists who are sent to serve in many local government health facilities across the country [57]. Desired capacities for local governments may then include having adequate financial resources to hire the cadres and quantity of health workers which their population requires, and to provide the full range of salaries and benefits, including security of tenure, for these health workers. Local governments may also be capacitated further when they are granted with the regulatory authorisation to hire more health workers as deemed necessary (of note, the national government’s Commission on Audit currently puts a cap on the percentage of the budget that can be used for salaries of all local government personnel, combining health and non-health personnel, yet local health services would often require more personnel than what is allowed). Such capacities may be complemented by accountability mechanisms that include the central/regional levels requiring local governments that benefit from the deployment programme not only to provide counterpart support for the staff they receive (e.g. free housing or transportation allowance for the deployed health workers), but more so to carry out a medium- to long-term plan to prepare the necessary local budget adjustments to hire the required workforce on their own in the near future. Without such conditionalities, local governments may become constantly dependent on the national government for its health workforce needs. Other accountability mechanisms may include the national/central level taking responsibility for building the capacity of local government health workers across the country [58], as well as implementing a national policy that discourages local health workers from being partisan during local elections in order to insulate them from politicisation.

### Data monitoring and utilisation

Finally, decision space for data monitoring and utilisation was assessed as moderate because local governments are already primarily responsible for collecting health-related data at local levels and for transmitting these data to the regional and central levels for consolidation by the DOH. Nevertheless, experiences in the Philippines suggest that many local decision-makers perform data collection out of mere compliance and sometimes lack the capacities for utilising the data to initiate actions at local levels. Thus, local decision-makers may optimise their decision space for this function when their capacities in basic epidemiology and evidence-informed public health are enhanced so that they understand what the indicators mean and how they can translate the data into effective decision-making. Accountability mechanisms in this function can include the DOH at national or regional levels deploying its own data collectors to local governments to validate the data being reported, and also to accelerate the transmission of the data to the central level. Such a scheme was recently introduced in the Philippines through the DOH’s deployment of ‘public health associates’, who perform parallel data collection at local levels. Furthermore, the national/central level may consider publicising an annual ranking of local governments in terms of meeting selected target health outcomes in order to inform the population of the performance of the local politicians they have elected in office, and likewise maintain a reliable central electronic database through the Field Health Services Information System [59], which pools all health-relevant indicators from local levels and is essential for accurately assessing the state of the Philippine health system as a whole.

## Discussion and conclusion

The results we have presented here are part of our efforts to understand how to make decentralisation work for the health sector [20, 35], this time focusing on the synergy of decision space, capacities and accountability. These results offer several opportunities for adjusting the capacities at local levels and strengthening accountability mechanisms to promote good decision-making in the devolved health system of the Philippines. The Framework Method used to analyse our interviews has allowed us to compare the perspectives between central/regional and local levels of decision-making which, for some functions, may be contrasting views. For example, in planning, some of the decision-makers at local levels felt they had the flexibility to plan on their own but some of the decision-makers at central/regional levels expressed that local plans were not fully implemented (Additional file 1). By comparing varying perspectives, we have triangulated these views and aimed to obtain an overall assessment of decision space for each function that drew from a synthesis of multiple views. Consequently, our analysis indicates that decision spaces at local levels have been mostly moderate or narrow despite 25 years of devolution in the Philippines.

The Philippine experience suggests that the moderate-to-narrow decision spaces observed at local levels are less the result of the national/central level refusing to grant the space, but more an indication of local decision-makers having inadequate capacities to fully perform the functions they have assumed in the aftermath of devolution. It would then appear that a truly wide decision space at local levels cannot be achieved unless it is accompanied by expanding capacities and strengthening accountability mechanisms. It is important to emphasise that the goal of this paper was not to prove this synergy. Bossert’s study in Pakistan has already provided a quantitative justification of how expanding each of these three dimensions potentially leads to improvements in selected health outcomes [26]. Through a qualitative approach, we have been able to explore a number of specific policy considerations under the assumption that the synergy works. Several studies on decentralisation in other low- and middle-income countries have also concluded that decentralisation only grants the decision space, but its effective use by decision-makers at lower levels of the system will be realised only when their capacities are built, as was reported, for example, in Fiji [60] and in India [28]. Particularly for the function of planning and priority-setting, capacity-building can improve the use of decision space as noted in Tanzania [61], and enhance transparency and accountability as likewise noted in India [62] and again at district levels in Tanzania [63].

We have presented a list of desired capacities, organised as individual and institutional/organisational capacities, to help improve the delivery of care in a devolved health system. At individual levels, these include, among others, skills for strategic planning, management, priority-setting, evidence-informed policy-making and innovation in service delivery. At institutional levels, these desired capacities include, among others, having a multi-stakeholder approach, generating revenues from local sources, partnering with the private sector, and facilitating cooperation between local health facilities. In the context of decentralisation, the responsibility for building the capacities of local decision-makers and the local governments which they constitute remains with the national/central decision-makers (i.e. the DOH and PhilHealth in the case of the Philippines).

In all health sector functions, we have noted a significant amount of augmentation provided by the national/central government in the Philippines to fill in the gaps of the local governments, especially in resource-poor areas, to fulfil their mandate to deliver quality health services to the populations they serve. This continuing intervention from the national/central level indicates the importance of analysing not only the roles of local level decision-makers but also the evolving role that central decision-makers play in decentralisation. Studies in other low- and middle-income countries have also reported some forms of re-centralisation despite decentralisation, such as in financing in Kenya [31], in ensuring equity in the distribution of physicians and health facilities in Indonesia [64], and in logistics systems or management of supplies in Ghana and Guatemala [65], where the evidence indicates the importance of having a combination of decentralised and centralised functions for optimal performance. With rapid transitions to decentralisation, similar to what happened in the Philippines, there have been similar disruptions in procurement resulting in drug stockouts in Kenya [32], as well as variations in the salaries of the health workforce resulting in strikes and mass resignations also in Kenya [32] and affecting the retention of primary care workers in rural areas in Nigeria [66]. These examples highlight, all the more, the important function of the central level to enforce accountability and shepherd the entire system as a whole to minimise such disruptions even as the system remains decentralised overall.

While there are multiple aspects to accountability, herein, we have focused on accountability mechanisms that the national/central level could enforce in a decentralised health system. The mechanisms we have enumerated encompass the aspects of financial (i.e. accounting the allocation and use of resources), performance (i.e. meeting the targets) and political (i.e. publicising the performance of local governments to inform voters) accountabilities as described in the introduction of this article. These accountability lines that link local decision-makers to national/central decision-makers offer opportunities for rewarding satisfactory performance of local governments with incentives and discouraging negligence in decision-making through regulation (i.e. a ‘carrot and stick’ approach), and our results present current mechanisms that could be enhanced, as well as potential policy considerations in the Philippines based on the perspectives of the decision-makers we have interviewed.

These insights from the Philippines draw from a growing realisation to move beyond linear causation towards more complexity-informed thinking [67]. Particularly for the Philippines, the solution to the challenges in its health sector may not come from either recentralising the health sector once more, or leaping towards federalism, but potentially from focusing on enhancing capacities and accountability regardless of what the governance structure of its health sector may be. By moving beyond an analysis of decision space alone through the lens of this synergy, we have explored opportunities for optimising decision-making at local levels with a four-step approach that first identified the decentralised function, then qualitatively analysed the decision space at lower levels for each function, and assessed the capacities and accountability adjustments required to improve decision-making within each function. This way of proceeding was useful to capture the complexity of analysing decentralisation and to yield concrete policy actions to be considered for the Philippines. Further studies on the Philippines may also explore the experiences of devolution for other sectors (e.g. agriculture, social welfare, public works, tourism, etc.) and compare lessons learned. In other words, to optimise decentralisation for the health sector, decision space at lower levels should indeed be widened, without forgetting to expand capacities and strengthen accountability. Bossert himself wondered if the synergy that he has demonstrated in Pakistan would also work in other settings [68]. It will be equally interesting and useful for policy, using a combination of methods, to see what recommendations emerge in analysing decentralisation in other settings using the lens of this synergy in order to truly optimise decentralisation for the health sector in the Philippines and other countries.

## Additional file

Additional file 1: Selected illustrative quotes extracted from the interviews, analysed using the Framework Method, and which provided basis for assessing decision space for each function as wide, moderate or narrow. The assessed decision spaces are linked to the dimensions of capacity and accountability in Tables 3 and 4 in the article. (PDF 211 kb)

## Abbreviations

DOH: Department of Health
LHB: Local Health Board
PhilHealth: Philippine Health Insurance Corporation
RHU: Rural Health Unit
