# Supplementary material for: Optimising decentralisation for the health sector by exploring the synergy of decision space, capacity and accountability: insights from the Philippines
Source: Health Res Policy Syst. 2019 Jan 10;17:4. doi: 10.1186/s12961-018-0402-1 (PMC6327786; doi:10.1186/s12961-018-0402-1)
Supplement: Supplementary file 1 — Selected illustrative quotes extracted from the interviews, analysed using the Framework Method, and which provided basis for assessing decision space for each function as wide, moderate or narrow. The assessed decision spaces are linked to the dimensions of capacity and accountability in Tables 3 and 4 in the article. (PDF 211 kb) [file 12961_2018_402_MOESM1_ESM.pdf]

Additional File 1: Selected illustrative quotes extracted from the interviews that were analysed using the Framework Method that provided basis for assessing decision space for each function as wide, moderate or narrow. The assessed decision spaces are linked to the dimensions of capacity and accountability in Tables 3 and 4 in the article.

<table>
<tr><th colspan="2">A. Planning</th><th>Comments</th></tr>
<tr><td rowspan="4">Local Levels</td><td>Municipal Health Officer (MHO) who is also a member of the Association of MHOs of the Philippines (AMHOP), 16 years in government:
“My staff and I plan to prioritise programs with low accomplishments. We then present the plan to the Local Health Board (LHB), chaired by the mayor who will approve it. We then meet with the other municipal health officers and present the plans to the province for consolidation. The planning process is okay, but would be better if we expand participation to involve more stakeholders from the Barangays [villages] and not only the local officials. Perhaps what is also lacking is the back-up. For example, the plan indicates that funding for this component will come from this agency, and then later the program does not receive the commitments, and the plan will not be implemented.”</td><td rowspan="5">These anecdotes from multiple backgrounds and levels of governance indicate that local decision-makers are indeed able to make decisions related to planning. Thus, decision space could not be seen as narrow. However, from development of the plans, to priority setting, involvement of stakeholders, and implementation of the plans, it is a common theme that local decision-makers cannot make decisions on their own without relying on the assistance, mostly technical in nature, provided by the (DOH) at central and regional levels. Thus, decision space cannot also be seen as wide. On the basis of the themes that have emerged, decision space for planning is therefore assessed as <u>moderate.</u></td></tr>
<tr><td>Mayor of a low-income municipality who is also a medical doctor, 26 years in government:
“National government wants LHBs to be functional, but it’s up to us to make it functional. We meet for the municipal health action planning, which flows from the Barangay health action planning. So the municipal plan is a consolidation of the various Barangay plans. The DOH has a representative in the LHB, and that is very good because the mayor doesn’t know everything. It’s a coincidence that the mayor here is a doctor, but how about those areas whose mayor is not a doctor? We need help from the DOH for the technical aspects, for example, in the family planning programme, immunisation, etc. We also review our shortcomings. But, you know, it varies from one municipality to another [laughs]. That is the disadvantage of devolution, right? The way things are is not uniform and depends on municipal leadership.”</td></tr>
<tr><td>City Health Officer (CHO) of a highly-urbanized city, 32 years in government:
“We have the minutes of the LHB meetings, but the attendees were actually not there! [laughs]. Kidding aside, what the national government wants now is to re-organize the LHB. During our last meeting with national government representatives to discuss good local governance, we were told to expand LHB membership. If I would really assess it, and I am free to say this, our LHB is not fully functional because there were times when we set a meeting and the mayor, who should chair it, was not around. Although it is possible for somebody else to be the presider, of course what we want is for the mayor to be there. So going back to that meeting, we were told that LHB functionality will now be part of the criteria for recognizing local governments with the seal of good local governance.”</td></tr>
<tr><td>Provincial Health Officer (PHO) who is also a member of the PHO Association of the Philippines (PHOAP), 32 years in government:
“DOH asks local governments, especially the provinces, to draw our local investment plans for health. DOH gives us the roadmap, guiding us where we should go, and then we in the province make a comprehensive plan containing what we need to do to improve our health system. In this plan we see the gaps that the municipalities are not able to address, which we in the province then pass on to the DOH. In other words, there is re-integration, in a way, between the DOH, the province, and the municipalities within it, integration which was present before devolution in 1992. We try to regain that integration through planning. And we still go back to what we consider our mother unit, the DOH, because it’s the DOH that can help local governments to enhance the operations of their health facilities.”</td></tr>
<tr><td>Central Level</td><td>Former Regional Director of the Department of Health (DOH), 29 years in government:
“Provincial and municipal plans are nice to read, but these are not followed [laughs]. We do so many workshops where someone from the DOH central office goes down and explains to local governments how planning should be done. Then we have issues with prioritisation during implementation. I really hold the local governments accountable. I say, ‘Your plan has not been completed yet, why then will the DOH give you additional funds?’ Something like that. The idea is nice once again, but there is so much political intervention, you know. There is lobbying up there, for example, in Congress where the budget is approved. You’ll be surprised that an item in the plan was slashed, or an item was included which we did not request for, all because of political interventions. How I wish that we really follow the plans.”</td></tr>
</table>

<table>
<tr><th colspan="2">B. Financing and Budget Allocation</th><th>Comments</th></tr>
<tr><td rowspan="4">Local Levels</td><td>MHO of a low-income municipality, 17 years in government:
"It's really difficult when we don't have money. When I started here in 2000, the annual budget for health for the entire municipality was only PHP 300,000 (USD 5,600). How can you possibly function with that? I asked our municipal council to increase it, but they could not approve it because the budget was already in place. In 2003, instead of using the budget to buy medicines, we decided to use it to enroll our constituents in PhilHealth. At that time, the premium was PHP 120 (USD 2) per family per year, and in return PhilHealth would pay us a capitation fund of PHP 300 (USD 6) per family after we provide primary care services to these families. In the end, we had something like PHP 600,000, and I could use half of that to buy medicines."</td><td rowspan="5">Drawing from the themes emerging from these interviews of various decision-makers, it appears that local decision-makers are somewhat able to make decisions related to local budget allocations and creation of other sources of financing, but only because of a significant amount of assistance coming from central sources (i.e. DOH support and PhilHealth payments). Otherwise, local decision-makers would likely be unable to make decisions in financing and budget allocation optimally when left on their own and without some form of central augmentation. Decision space for this function is therefore assessed as <u>moderate-to-narrow.</u></td></tr>
<tr><td>PHO of a high-income province, 21 years in government:
"About 25-27% of our Internal Revenue Allotment (IRA) is allocated for our hospitals, and about 5-7% for preventive services. I have an income recovery scheme here. The province provides the budget for maintenance and other operating expenses of hospitals, but I tell the hospitals to recover at least 90% of that and return the funds to the province. The hospitals are able to recover it through their PhilHealth income, and also through income from services not covered by PhilHealth but outpatients pay for, such as ultrasound or CT scan. So majority of our local budget is used for hospital operations, and that's curative, right? That means we spend so little for preventive services, which should have a bigger investment. This is what I want to ask from DOH, to provide additional funding to enhance our delivery of public health programs."</td></tr>
<tr><td>Mayor of a highly-urbanized city, 19 years in government:
"In 1998, when I was Congressman, I would ask the DOH during budget hearings to provide funds to the district hospitals in my province. And the common answer from the DOH was that these facilities were already devolved to the provincial government, and the DOH could not provide anything. When I became Governor, I enrolled my constituents in PhilHealth using the provincial budget. I would say that one of the key elements of success was PhilHealth. In fact, there was a time when we had the biggest enrolment in the country, more than 300,000 PhilHealth members. In the beginning, I knew that our provincial and district hospitals could not yet cope with the increasing demand from the increase in enrolled members. But I said that was okay, and we would be sustaining the financial operations of our hospitals."</td></tr>
<tr><td>Former Provincial Governor, 17 years in government:
"What is lacking? Responsibilities have been given, granted. But resources continue to be centralised. The sharing of taxes is still heavily in favour of the national government, 60-40. The Local Government Code says that the regional offices of national agencies whose functions have been devolved should be diluted into monitoring offices providing technical assistance. The reality is, they continue to function, and the budget of the DOH continues to increase through the years. Now, local governments should not be dependent on their share from national taxes called IRA. They also have their local taxing powers to raise their own revenues. But local governments are not on equal standing. Some are industrialized, some are agricultural. Some have real property. So the funds that local governments could raise are not enough to underwrite the expenses for the devolved services. And Congress will not want that national funds are diminished because they won't have access to the funds if these are downloaded to local governments."</td></tr>
<tr><td>Central Level</td><td>Former high-level official of the DOH Central Office, 41 years in government:
"The important thing is for local governments to understand. Once I visited a province, and I saw that their new maternal delivery unit had been completed. How many deliveries have been performed here? I asked them. They showed me the list, and it was around 30 per month. Is this facility accredited by PhilHealth? I asked. They said not yet, and they were passively waiting for the outcome of their application for accreditation. And they have been operating for 3-4 months already. I showed them the computations. They were missing like PHP 800,000 (USD 15,000) worth of potential PhilHealth payments in the last four months. Do you realize that this is the money you are missing? I told them. Even the Governor was surprised. I told him, now that you know, would you be more pro-active in securing accreditation?"</td></tr>
</table>

	C. Programme Implementation and Service Delivery	Comments
Local Levels	**MHO of a high-income municipality, 12 years in government:**<br>*"The DOH changed the EPI [Expanded Programme on Immunisation] and shifted to Td [low-dose diphtheria toxoid] as Tetanus vaccine. Oh, I was not aware of it, and the new vaccines just arrived here. So I called the DOH requesting for guidelines. My wish is for us to be informed well. Are they really asking us down here? The programme just goes down. It's a pity for the MHO because I have to dance with all of these programmes. But I do have the freedom to delay implementation, because I also need to think about how these programmes fit our municipality. But I cannot really disobey because it would be embarrassing as the DOH supports us in so many ways. If we don't comply, we are like ungrateful. So all programmes are coming from the DOH, but we modify in implementation. For example, DOH wants us to implement an NCD [non-communicable disease] programme and form NCD clubs. But we already have a diabetes club here. So I just expanded the diabetes club."*	Analysis of the interviews of decision-makers indicates a variation in perspectives on decision-making related to programme implementation and service delivery. On the one hand, implementation of health programmes by the local governments is largely the result of what is being cascaded down by the DOH from central and regional levels. On the other hand, local governments also have some flexibility in deciding how to implement such programmes at their level. For these reasons, decision space is assessed as **moderate.**
	**Assistant CHO of a highly-urbanized city, 22 years in government:**<br>*"The DOH has a role because there are issues which we cannot answer as a city. For example, during our last maternal and child health programme meeting, a question was raised about HIV-positive pregnant women who deliver in our birthing facilities. We do not have guidelines on how to manage the newborn from an HIV-positive mother. So we go back and ask the DOH because the technical guidelines should come from them. If the mother has hepatitis B, how do we care for the baby? And we do not have immunoglobulin here for the baby. The DOH should provide the guidelines for that. On Tetanus toxoid, the DOH decided suddenly to change that and buy Td instead. Can we give that to pregnant women? They should give us the guidelines for that. If something wrong happens to our patient here, we have the DOH guidelines to show for it. I know that it takes time to produce guidelines. But we cannot also neglect our patients."*	
	**Member of Congress representing a low-income province, 26 years in government:**<br>*"What I don't like about devolution is sometimes the department heads no longer have an initiative to think about their own programmes. It's like they always depend on the national government. Because that's their comfort zone, to follow whatever DOH says up there. But sometimes it is really good that you have your own programme in your locality which you could truly call your own. Then if you would see that there are national programmes which you think are appropriate to the local context, then apply these. Otherwise, what is happening is that the PHO would insist to convene all of these committees, such as an HIV/ADS council for example, because that is what is being prescribed by the DOH from above, and we have all these committees which may not be relevant to our problems here."*	
Central Levels	**Regional Director of the DOH, 32 years in government:**<br>*"It's not so difficult to implement DOH programmes at local levels especially if we're able to engage the politicians. It's really engaging them as the strategy. Once we are able to establish the relationship with the local government, it would be easy. For my part, I do it personally, by making phone calls to the governors and mayors. When the DOH rolled out the MR-OPV (measles, rubella, oral polio vaccine) mass immunisation in 2014, this region achieved 91% coverage and vaccinated more than a million children. If we got a low accomplishment, national accomplishment would be pulled down as we are a big region. So I made calls to the governors and mayors, personally updated them, and made strategic visits also to about 15 Barangays. I did what it meant to be politically-savvy. It's really letting them realise that they are part of the solution."*	
	**High-level official of the DOH Central Office, 28 years in government:**<br>*"As the devolution process evolved, and as local governments become more capable to handle their health services, there were circulars issued by the DOH programmes in the central office to ensure quality, for example, on how to package the tuberculosis control programme for their locality. Some of these guidelines sought to remedy the negative aspects of devolution, and so the concept of interlocal health zones or service delivery networks to group local governments together emerged to encourage different local governments serving the same catchment area to deliver health services in a harmonised manner."*	

<table>
<tr><th colspan="2">D. Management of Facilities, Equipment and Supplies</th><th>Comments</th></tr>
<tr><td rowspan="3">Local Levels</td><td>MHO of a low-income municipality, 16 years in government:
“If I compare this municipality with the other GIDA [Geographically Isolated and Disadvantaged Areas] municipalities, we have the most number of BHS [Barangay Health Station] constructions because of HFEP [Health Facility Enhancement Programme]. So as MHO, I proposed these to the DOH because I could see that we do not have a physical structure when I visit the barangays. Sometimes, we deliver services under the tree, sometimes in the Barangay (village) hall, sometimes in the school. So for every Barangay there is now a BHS. We also have equipment now because HFEP includes the corresponding equipment and instruments for every structure built, and the counterpart of the local government is to provide the staff.”</td><td rowspan="5">The themes emerging from decision-making related to management of facilities, equipment and supplies suggest that most local governments in the Philippines have adequate latitude to decide on how they manage these resources, but are fully able to do so if with assistance from DOH and PhilHealth and, in some cases, private sector involvement. This observation is noted across decisions related to what facilities to build, how to maintain these facilities, and how to make these fully-equipped. Decision space for this function could neither be wide nor narrow and, thus, the overall assessment of <u>moderate</u> is made.</td></tr>
<tr><td>DOH-hired medical doctor deployed to serve as MHO (“Doctor to the Barrios” program), one year in government:
“It’s really DOH that implements the construction for HFEP. For example, I will receive a letter from the DOH saying that budget for HFEP 2018 is available, and these are the eligible projects, health facilities, or medical equipment. So whatever we need for facilities and equipment we would already state there in the proposal, which we submit to the DOH regional office. And then we wait until there is news of approval. Actually, I just wait [laughs] until it is finished. So this new facility we have, I really pushed for it. Because when I talk to the contractor, he would say that they have not yet been paid by the DOH. But when I talk to the DOH, they would say that they have paid the contractor already. So I don’t know anymore, but we already need the facility.”</td></tr>
<tr><td>PHO of a high-income province, 21 years in government:
“The local government did not really have the money to buy huge equipment. I advised the Governor to get into public-private partnerships. We engaged companies that would place diagnostic machines in our hospitals, like CT scan and ultrasound. We told the companies that we would not buy the equipment but they would lend it to us under an agreement, and they get a monthly payment from us for using their equipment. We discuss a recovery scheme, which should not compromise the income of the province. For example, with our X-ray machines, the patient pays PHP 300 (USD 6), 100 of that goes to the private partner, and 200 goes to the province. We tell our private partner that their advantage is that they are securing not only one hospital but 14 hospitals owned by the province. On the other hand, I tell the Governor that the partnership is good for the province because we will not shell out a huge amount of money from our IRA to buy equipment which is just being leased to us, but we are still able to provide the service to our constituents. Maintenance and upgrade of the equipment will also be the responsibility of the private partners.”</td></tr>
<tr><td rowspan="2">Central Levels</td><td>Director in the DOH Central Office, 28 years in government:
“If you would look at how the DOH works with local governments now, it seems that a bulk of our budget actually goes to them. It’s as if it is not devolved. During the last years, DOH upgraded their facilities. DOH is also providing the commodities for the programmes. DOH is giving them the drugs, TB drugs, and now even hypertensive drugs, diabetic drugs. So there is always that question, are we really in a devolved set-up? It has been observed that the local governments really do not have the capacity for health services. I’m not saying that this is happening across the country, but in most municipalities and provinces, most especially in the low-income ones, well, even in some first-class provinces. Why? Because the population has increased but there was no increase in the infrastructure and the personnel. That’s why the DOH augments the local governments.”</td></tr>
<tr><td>High-level official of the Philippine Health Insurance Corporation (PhilHealth), 20 years in government:
“The RHUs [Rural Health Units] of the municipalities can be accredited by PhilHealth as maternity care provider, or primary care package provider, or TB-DOTS package provider. So we have to make sure that, because these facilities are not licensed like how DOH would license hospitals, the RHUs would have to go through inspection by our accreditation teams to be eligible for PhilHealth payments. So the RHUs must meet the standards. Well, some municipalities do not have enough budget to make sure that they pass the accreditation not only because they are poor, but because the services are not complete. For example, an RHU cannot be accredited as maternity care provider if there is no midwife, or no obstetrician for referral, or they lack equipment and medicines.”</td></tr>
</table>

<table>
<tr><th colspan="2">E. Health Workforce Management</th><th>Comments</th></tr>
<tr><td rowspan="3">Local Levels</td><td>Mayor of a middle-income municipality who is also a lawyer, 21 years in government:
“At the moment, we really lack health personnel because we don’t have the capacity to hire. In fact, just recently, we increased the salaries of personnel, as required by law, because the rates of health personnel are higher than those of other local employees. This is where we have a problem. We now have a population of 42,000 but we only have one doctor in the Rural Health Unit. It is also not possible for us to just hire and hire because there is this PS [personnel salaries] cap set by the Commission on Audit that limits what we can use for salaries to a certain percentage of our budget. We already proposed that we will pay personnel with honoraria rather than salaries because in this way we can contract additional personnel.”</td><td rowspan="5">The themes that emerge from the perspectives at various levels of governance indicate that local decision-makers are able to hire the health workforce they need, but in many cases decisions are limited by the lack of resources to provide salaries and also by political considerations. Some local decision-makers have also expressed dissatisfaction with the variations in their compensation, lack of opportunities for career development, and the frequent intervention of politicians in decision-making for health workforce management. While the DOH has aimed to augment the lack of health workforce in some localities through the deployment of centrally-hired personnel to these areas, it remains to be seen if this strategy is sustainable and would result in the availability and retention of the health workforce in the long-term. Given this context, decision space is assessed as <u>moderate-to-narrow.</u></td></tr>
<tr><td>MHO of an island municipality, 16 years in government:
“In hiring, of course, we consider political favors. If the applicant is not from the same party as the mayor’s, then he/she will not be hired. Even if I like this applicant who is very capable, but if he/she supported a different party during the last elections, he/she will not be hired. We cannot do away with this in the Philippines. You know, I have worked in the local government for so long, and I have learned to embrace politics and dance with the situation. My efforts will go to waste if I fight for the applicant I want and then I have conflict with my mayor.”</td></tr>
<tr><td>PHO of a low-income province, 29 years in government:
“A poor province could only afford this much, and cannot provide salaries like a wealthy province could. In my case, my salary is only for a second-class province, because my province has the capacity of only a second-class local government. If the province becomes first-class, then the salaries will go up too. That is why when you compare the salaries in different classes of provinces or municipalities across the country, the rates would be different. I think salaries should be standardised across the country, regardless of where one is serving, because we are all doctors anyway, same with nurses and midwives. We are all health professionals, right?”</td></tr>
<tr><td rowspan="2">Central Levels</td><td>Representative of a non-government organization (NGO) assisting local governments, 14 years in government:
“I think the deployment of health workers by the DOH to augment the staff in local government facilities is good. But, wait, what do they actually do there? Okay, so they send 20 nurses over there, but they sit there in the facility. I think augmentation is needed but it should not be done every year. It has to be rational. There are human resources provided by the center, but how are those resources effectively used at the local level? It’s like, oh, this local government needs five midwives, so I give it five midwives. And then the other local government says, we were given 10 but we do not know what to do with them [laughs].”</td></tr>
<tr><td>Former high-level official of the DOH Central Office, 23 years in government:
“From the point of view of health personnel, it would really be better if they were part of one organisation. Because from the point of view of career, somebody who does well in a local government can be tapped by the DOH, and can be assigned in different places, giving a lot of flexibility for that person’s career. Under devolution, you know, you work in a province or municipality, and you want to move to another province, or work at the regional level, or in the central office—it’s much more difficult to move because these are not the same organisations. And then, the central level will not be looking necessarily at people in the provinces or municipalities for new blood to reform the organisation because the local governments are not under it. I mean, DOH would not have the data at their fingertips, unless they’ve actually developed an information system that could tap deep into local government files.”</td></tr>
</table>

F. Data Monitoring and Utilisation		Comments
**Local Levels**	**MHO of a high-income municipality, 12 years in government:**<br>*"What's happening now with the DOH's deployment of the PHAs [public health associates] to us is that the PHAs directly interact with the DOH for the data reports. So from the municipality, sometimes I don't even see the reports anymore, reports go directly to the DOH regional office. The PHO is bypassed and has lost its role. Ideally, the DOH should ask the data from the PHO because the municipalities submit the reports to the province. Monitoring is also too programmatic. Each DOH programme coordinator also asks us for data relevant to their programme only. Ideally, all these reports, for whatever programme there is, should be consolidated in a database already. We are also still paper-based. We should be electronic already, but different local governments use different EMRs [electronic medical records]. We are waiting for the interoperability of these EMRs."*	The themes from the interviews reveal contrasting views on how decisions related to data monitoring and utilisation are made between those at central/regional and those at local levels. Nonetheless, from the point of view of local decision-makers, the collection of indicators and management of data is mostly within their purview, while the DOH performs the function of ensuring the timeliness, accuracy, consolidation and utilisation of the data. For these reasons, decision space is assessed as **moderate.**
	**Assistant CHO of a highly-urbanized city, 22 years in government:**<br>*"Perhaps if you ask the DOH, they would tell you that they are having a hard time with the data because of devolution. It takes a long time for us to submit reports to them. Why am I taking a long time? For my part, I am consolidating all of the reports, including those from our hospitals. So, it is difficult, right? Oh, we do our own surveillance and DOH also does its surveillance, that's why it is difficult. Actually, there are instances when DOH detects cases first before we do. And there was a time also when we detected it first before they did. So before the DOH even learns about it, we already have a report. That is why maybe for them the DOH is saying that it's more difficult. Because they feel there is an extra step before the data gets to them, and the city still needs to gather the data from all our health centers."*	
	**PHO of a low-income province, 29 years in government:**<br>*"We are able to complete the data for the province, usually until after the end of the year, around the last week of February or first week of March. When we perform data quality checks, we would really see that it is not perfect, we would see many problems with the data. We are still paper-based. Although there is also an electronic database, but then usually from down there it is paper-based and then the data is manually transferred to the computer in our office. If we want to check the data, what we examine is the hard copy, because maybe there was a problem in the encoding. But from my view, this is already improving."*	
**Central Levels**	**Regional Director of the DOH, 32 years in government:**<br>*"It's difficult to collect data because even with a digital database, local governments submit reports late. So we install our own data collectors called PHAs in local governments to do parallel data collection. Sometimes the PHO complains because local data are transmitted directly to the DOH regional office. These are issues we need to be aware of to ensure coordination, but the DOH also cannot just wait always for the delayed data from the local governments when we need to make decisions."*	
	**Regional Director of the DOH, 34 years in government:**<br>*"From the municipality, the reports go to the province, although the DOH regional office is furnished with a copy. It's the province that consolidates the reports and submits to the region. And then we consolidate it and submit it to the central office. We are now reviewing the process for the data to be more current, because that is how it should really be, that we have the data we need to act at this time without having to wait for consolidation. Now we have what is called a dashboard where we have certain indicators to look at. When we had problems with the maternal health programme, we were collecting maternal indicators monthly through our deployed staff and did not have to wait for the local governments, so that we already know the trends and could act even before the official report is finalised. What is good about the dashboard is that with certain indicators we can compare areas, so we have an idea how far we are from our targets. Previously, to be truthful about it, the local governments were already estimating in advance what they would accomplish, and that is what they were reporting."*	
